# Supplementary material for: Sorafenib as an Inhibitor of RUVBL2
Source: Biomolecules. 2020 Apr 14;10(4):605. doi: 10.3390/biom10040605 (PMC7226205; doi:10.3390/biom10040605)
Supplement: Supplementary file 1 [file biomolecules-10-00605-s001.zip › Figure S1_SoraAnalogs_120319.pdf]

Analog 1	Analog 2	Analog 3	Analog 4
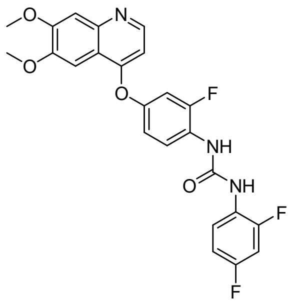	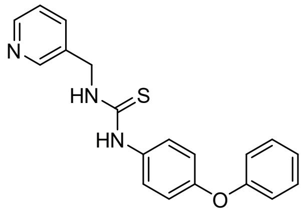	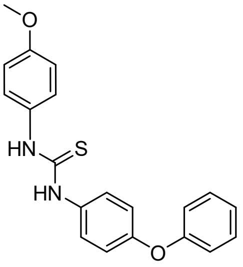	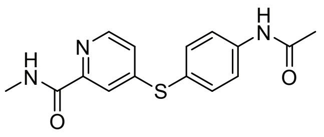
Analog 5	Analog 6	Analog 7	Analog 8
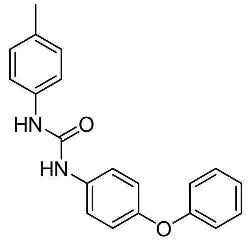	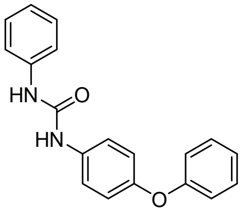	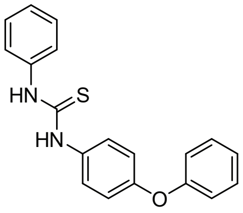	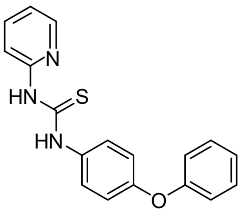
Analog 9	Analog 10	Analog 11	Analog 12
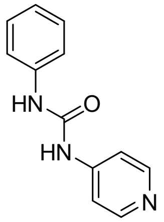	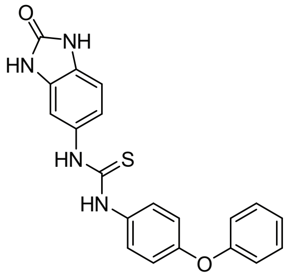	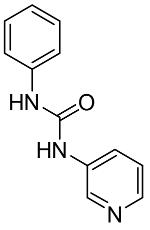	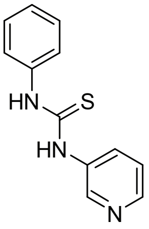
Analog 13	Analog 14	Analog 15	Analog 16
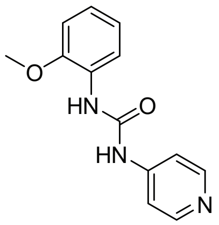	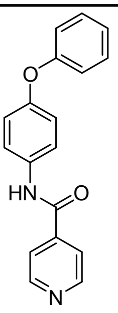	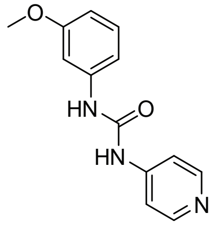	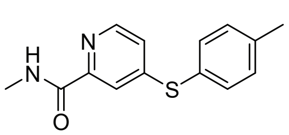
