# Supplementary material for: Sorafenib as an Inhibitor of RUVBL2
Source: Biomolecules. 2020 Apr 14;10(4):605. doi: 10.3390/biom10040605 (PMC7226205; doi:10.3390/biom10040605)
Supplement: Supplementary file 1 [file biomolecules-10-00605-s001.zip › Supp_Tables_121419.docx]

**Table S1. List of proteins, plasmids and expression strains used in this study.**

| Protein | Plasmid | Tag | *E.coli* strain used for expression | Protease used for tag cleavage | Source |
| --- | --- | --- | --- | --- | --- |
| RUVBL1 WT | Profinity eXact pPAL7 | N-terminal Profinity eXact | BL21(DE3) pRIL | Subtilisin | Current study |
| RUVBL1 WT | p11 | N-terminal His_6_-TEV | BL21(DE3) pRIL | TEV | Current study |
| RUVBL1 WB | Profinity eXact pPAL7 | N-terminal Profinity eXact | BL21(DE3) pRIL | Subtilisin | Current study |
| RUVBL2 WT | p11 | N-terminal His_6_-TEV | BL21(DE3) pRIL | TEV | Current study |
| RUVBL2 WB | p11 | N-terminal His_6_-TEV | BL21(DE3) pRIL | TEV | Current study |
| RUVBL2 ND | p11 | N-terminal His_6_-TEV | BL21(DE3) pRIL | TEV | Current study |
| RUVBL2 DN | p11 | N-terminal His_6_-TEV | BL21(DE3) pRIL | TEV | Site-directed mutagenesis |
| RUVBL2 NN | p11 | N-terminal His_6_-TEV | BL21(DE3) pRIL | TEV | Current study |
| RUVBL2 ΔDII | pET21 | N-terminal His_6_-TEV | BL21-Gold(DE3)pLysS | Not cleavable | Generous gift  from Dr. I. R. Tsaneva  (Niewiarowski et al. 2010, *Biochem J* 429, 113-125) |
| RUVBL2 WT-RUVBL1 WT | pCOLADuet-1 | eXact tag-RUVBL2/  RUVBL1-TEV-His_6_ | BL21(DE3) pRIL | Subtilisin and TEV | Current study |

**Table S2. List of mutations and primers used for subcloning and mutagenesis in this study.**

| Plasmid-Gene names | Mutation | Primers | Sequences |
| --- | --- | --- | --- |
| pPAL7-RUVBL1 WT | None | RUVBL1 HindIII F | GGCGAAGCTTTGATG AGATTGAGGAGGTGAAG |
|  |  | RUVBL1 BamHI R | CCGGATCCTCACTTCATGTACTTATCCTGC |
| pPAL7-RUVBL1 WB | D302N | RUVBL1 WB D302N F | GCTGTTTGTTAATGAGGTCCAC |
|  |  | RUVBL1 WB D302N R | GTGGACCTCATTAACAAACAGC |
| P11-RUVBL1 WT | None | RUVBL1 NdeI F | CAGGGCCATATGAAGATTGAGGAGGTGAAG |
|  |  | RUVBL1 BamHI R | CCGGATCCTCACTTCATGTACTTATCCTGC |
| p11-RUVBL2 WT | None | RUVBL2 NdeI F | CAGGGCCATATGGCAACCGTTACAGCCAC |
|  |  | RUVBL2 BamHI R | CCGGATCCTCAGGAGGTGTCCATGGTC |
| p11-RUVBL2 WB | D299N | RUVBL2 WB D299N F | CTGTTCATCAACGAGGTCCAC |
|  |  | RUVBL2 WB D299N R | GTGGACCTCGTTGATGAACAG |
| p11-RUVBL2 ND | D349N | RUVBL2 D349N F | GGCATCCCCATAAACCTGCTGGACCGG |
|  |  | RUVBL2 D349N R | CCGGTCCAGCAGGTTTATGGGGATGCC |
| p11-RUVBL2 DN | D352N | RUVBL2 D352N F | gacctgctgaaccggctgcttatc |
|  |  | RUVBL2 D352N R | gataagcagccggttcagcaggtc |
| p11-RUVBL2 NN | D349N/ D352N | RUVBL2 D349N/352N F | GGCATCCCCATAAACCTGCTGAACCGGCTG  CTTATC |
|  |  | RUVBL2 D349N/352N R | GATAAGCAGCCGGTTCAGCAGGTTTATGGG GATGCC |
| pCOLADuet1-RUVBL2 WT-RUVBL1 WT | None | Profinity eXact tag  NcoI F | GAACcatgggagggaaatcaaacg |
|  |  | Profinity eXact tag BamHI R | GAAAGGATCCCAAAGCTTTGAAGAGC |
|  |  | RUVBL2 BamHI F | GAGGggatccatggcaaccgttacagc |
|  |  | RUVBL2 Afl II R | CAACTTAAGTCAGGAGGTGTCCATGGTCTCGC |
|  |  | RUVBL1 NdeI F | CAGGGCCATATGAAGATTGAGGAGGTGAAG |
|  |  | RUVBL1-TEV seq-6x His KpnI R | CAAGGTACCTCAATG ATGATGATGATGATGAGC AGCGCCCTGGAAATACAAGTTTTCCTTCATGTACTTATC |
