# Supplementary figures and images for: Sorafenib as an Inhibitor of RUVBL2
Source: Biomolecules. 2020 Apr 14;10(4):605. doi: 10.3390/biom10040605 (PMC7226205; doi:10.3390/biom10040605)

A

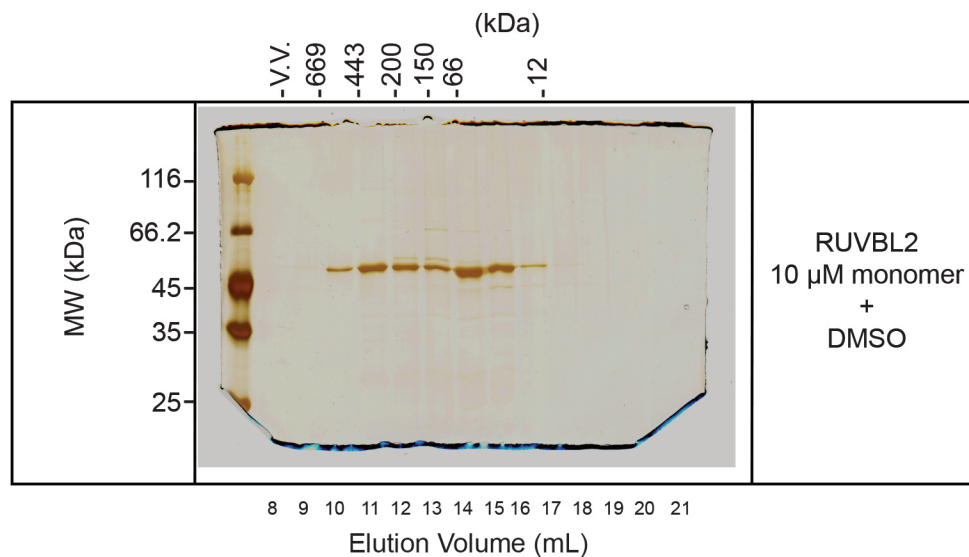

B

|                   |   |   |   |
|-------------------|---|---|---|
| 30 $\mu$ M RUVBL2 | + | + | + |
| DMSO              | - | + | - |
| 87 $\mu$ M Sora   | - | - | + |

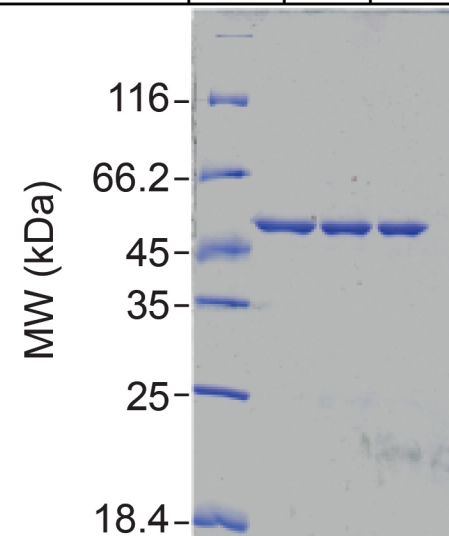

Supplement: Supplementary file 1 [file biomolecules-10-00605-s001.zip › Figure S2_FullGels_010719.pdf]
